# Consumer Acceptability, Eye Fixation, and Physiological Responses: A Study of Novel and Familiar Chocolate Packaging Designs Using Eye-Tracking Devices

**DOI:** 10.3390/foods8070253

**Published:** 2019-07-12

**Authors:** Nadeesha M. Gunaratne, Sigfredo Fuentes, Thejani M. Gunaratne, Damir D. Torrico, Hollis Ashman, Caroline Francis, Claudia Gonzalez Viejo, Frank R. Dunshea

**Affiliations:** 1 Faculty of Veterinary and Agricultural Sciences, School of Agriculture and Food, University of Melbourne, VIC 3010 Parkville, Australia; 2 Department of Wine, Food and Molecular Biosciences, Faculty of Agriculture and Life Sciences, Lincoln University, Lincoln 7647, New Zealand

**Keywords:** emotions, familiarity, biometrics, consumer liking

## Abstract

Eye fixations on packaging elements are not necessarily correlated to consumer attention or positive emotions towards those elements. This study aimed to assess links between the emotional responses of consumers and the eye fixations on areas of interest (AOI) of different chocolate packaging designs using eye trackers. Sixty participants were exposed to six novel and six familiar (commercial) chocolate packaging concepts on tablet PC screens. Analysis of variance (ANOVA) and multivariate analysis were performed on eye tracking, facial expressions, and self-reported responses. The results showed that there were significant positive correlations between liking and familiarity in commercially available concepts (*r* = 0.88), whereas, with novel concepts, there were no significant correlations. Overall, the total number of fixations on the familiar packaging was positively correlated (*r* = 0.78) with positive emotions elicited in people using the FaceReader™ (Happy), while they were not correlated with any emotion for the novel packaging. Fixations on a specific AOI were not linked to positive emotions, since, in some cases, they were related to negative emotions elicited in people or not even associated with any emotion. These findings can be used by package designers to better understand the link between the emotional responses of consumers and their eye fixation patterns for specific AOI.

## 1. Introduction

Food product development and innovation have been important strategies of food companies that compete within global markets [1]. However, despite these strategies, over 60% of failure rates are sometimes observed in the market [2]. Previous studies conducted by Nielsen [3] showed that new products require an optimal duration time of six to twelve months to be accepted in the market. When buying new products in the supermarket, consumers are generally unaware of the taste prior to purchase, and the aesthetic design and the visual appearance of the food packaging provides value to the food product and impacts their willingness to purchase [4]. A challenge commonly faced by food companies is how to distinguish/differentiate competitive products in the market [5]. In order to accomplish this, methods to attract consumer attention have always been a challenge for food companies [6]. 

A general assumption made by food package designers is that the consumers can read and interpret the information provided on the packaging [7]. However, consumers with low literacy and numeracy skills are likely to give up trying to understand the information provided on the packaging [7]. A review conducted by the Food Standards Agency stated that 38% of consumers from Australia and New Zealand found it hard to understand the information provided on food packaging [8]. Besides, only one in three consumers has the appropriate knowledge to understand the information on food packaging [9]. However, there have only been a few research studies conducted to understand how consumers would react when they are exposed to packaging that has many elements/signs, which requires attention to evaluate the food labels [10]. This makes the understanding of consumers’ gaze movements important, hence the implementation of eye-tracking sensors that capture the gaze fixations and offer an excellent opportunity to understand consumer behavior. Most of the studies conducted using packaging have been focused more on health claims [11,12,13] and labeling schemes [14,15]. Therefore, the assessment of the gaze movements of consumers may be novel and of great interest to the food industry.

Packaging is comprised of brand elements, texts, illustrations, regulated and unregulated signposts, content descriptions, and the packaging container [16]. These visual elements of packaging try to convey a valuable set of brand impressions similar to how consumers endorse advertising and pricing, and therefore the way these visual elements are presented to the consumer is very important [16]. In addition to these visual elements, consumers are exposed to competing products with an overwhelming number of visual stimuli in and around the shelves of supermarkets. The exposure to several elements in packaging and the exposure to competitor’s products make the decision-making process very complicated [17]. This information overload may cause disinterest and lower attention within consumers who could make inappropriate purchase decisions [10]. A large portion of human decision making is intuitive, impulsive, automatic, and unconscious [18]. These unconscious reactions need to be assessed in order to understand the decision-making process of consumers. 

The human eye is the sensory organ for the visual and cognitive system, and thus, gaze fixations are very important to direct visual attention to an object [19]. A study by Shepherd et al. [20] states that attention is not necessarily linked to the corresponding gaze movements, but it is impossible for gaze to move without a shift in attention. During the past decade, there has been mounting evidence based on eye tracking that could provide additional information to consumers’ behavior [21], since gaze movements are good behavioral indicators for measuring visual attention and information acquisition [22]. The unconscious responses governed by the autonomic nervous system (ANS) of the brain are based partly on gaze movements and on where people direct their attention. The human brain uses attentional mechanisms to select a subset of information when looking at a stimulus [23]. Thus, attention is important to the decision making of consumers [24]. 

Modern eye-tracking technology is used to measure gaze movements by combining a computer screen with an infrared camera to record pupil and corneal reflection in order to track their movements [10]. It has been used in many research studies related to reading and visual experience [11,12,25,26,27,28,29]. Most assumptions are made based on the idea that longer eye fixations are associated with higher levels of attention and interest, and greater interest equals positive reaction, liking, and assimilation [18]. However, eye tracking alone cannot provide accurate information in terms of the emotional responses of consumers [18]. Hence, this technology may be coupled with the measurement of other physiological responses that will help to understand consumer behaviors towards different products better. 

This study used eye-tracking technology combined with the assessment of facial expressions obtained from videos from participants translated into eight emotions, two dimensions, five facial states, head orientation (X-Y-Z), and gaze direction using the FaceReader™ (FR) software (Noldus Information Technology, Wageningen, Netherlands) [30]. The six novel packaging concepts used for this study were designed based on the TNS NeedScope model. This model is based on universal human emotions and is a foundation for understanding needs across cultures and markets. The framework is made up of six segments (fun, new, premium, healthy, ritual eating, and every day). Each segment has the following three layers: at the core is the archetype, the middle layer is the needs, and the outer layer is the emotions. The vertical axis (extroverted and introverted) represents the interaction with the outside world, while the horizontal axis represents the interaction with others (social and personal) [31]. This study aimed to assess the links between the emotional responses obtained by facial expressions from consumers with the eye fixations on areas of interest (AOI) of different chocolate packaging designs using eye trackers to better understand consumers’ perceptions of different packages.

## 2. Materials and Methods

### 2.1. Participants for Sensory Sessions

Panelists (*n* = 60, 32 females, 28 males) were recruited via e-mail invitations, with respondents between 25 and 55 years from the University of Melbourne, Australia who volunteered to participate in the sensory assessment of food packaging. According to the Power analysis, the number of consumers in this study (*n* = 60) was enough to find significant differences (1 − β > 0.999). This was calculated using SAS^®^ Power and Sample Size 14.1 software (SAS Institute Inc. Cary, NC, USA) for one-way ANOVA. This is in accordance with Gacula and Rutenbeck [32], who concluded that the sample size within the range of 40–100 consumers has enough power to find significant differences. The experimental procedure was approved by the Ethics Committee of the Faculty of Veterinary and Agricultural Sciences at the University of Melbourne, Australia (ethics ID: 1545786.2). Panelists were asked to sign a consent form before the experiments as per ethics requirements.

### 2.2. Stimuli 

**Figure 1:**
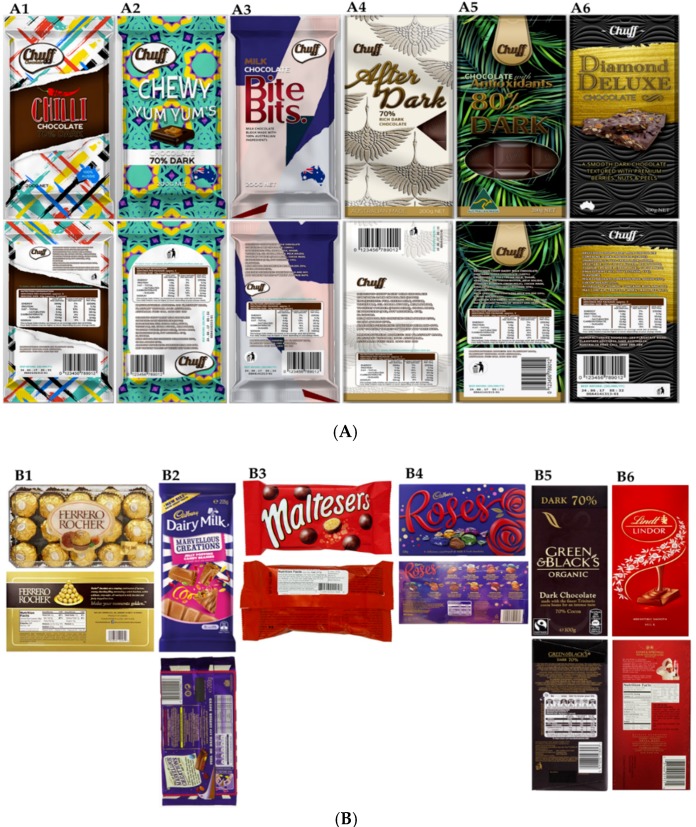
Front and back images of (**A**) novel and (**B**) familiar packaging concepts used for the study. (**A**) (A1) bold, (A2) fun, (A3) every day, (A4) special, (A5) healthy, and (A6) premium. (**B**) (B1) Ferrero Rocher, (B2) Cadbury Marvellous Creations, (B3) Maltesers, (B4) Roses, (B5) Green & Black’s, and (B6) Lindt).

Chocolate packaging was selected as the stimulus for this experiment since it is considered to be a type of food with health benefits and emotional satisfaction, which could be reflected in the emotional responses from panelists [33]. Novel chocolate packaging concepts were developed based on the six segments of the TNS NeedScope model (bold, fun, every day, special, healthy, and premium) [31]. As shown in Figure 1A, the front and back of the packaging for novel concepts (A1: bold, A2: fun, A3: everyday, A4: special, A5: healthy, A6: premium) were developed using the SolidWorks software (SolidWorks Corporation, Waltham, Massachusetts, USA) for each concept. The familiar packages (commercial), as shown in Figure 1B, were selected based on where brands were positioned representing the following six segments of the TNS NeedScope model: B1, bold (Ferrero Rocher, Ferrero S.p.A., Pino Torinese, Italy); B2, fun (Cadbury Marvellous Creations, Mondelez International, Uxbridge, London, United Kingdom); B3, every day (Maltesers; Mars Inc., McLean, VA, USA); B4, special (Roses, Mondelez International, Uxbridge, London, United Kingdom); B5, healthy (Green & Black’s, Mondelez International, London, England); and B6, premium (Lindt, Kilchberg, Zurich, Switzerland). These were obtained by a focus group discussion conducted with 30 participants. Three sessions were conducted with 10 participants in each session. The qualitative multivariate analysis (QMA) was used, and the participants were asked questions about food packaging, emotional attachment towards food, and how they fit the packaging elements to the six segments of the TNS NeedScope model [34]. Sixty different concepts and elements (ten for each segment) were shown, and one concept for each segment was selected. The QMA is a consumer research protocol which captures consumer insights in order to understand the link between important values of products. It has an advantage over conventional methods, such as focus groups and one-on-one interviews, because it minimizes bias [35].

### 2.3. Sensory Session, Video Acquisition, and Analysis 

For this study, we used individual sensory booths with uniform white light located in the sensory laboratory of the Faculty of Veterinary and Agricultural Sciences (FVAS) at the University of Melbourne. Each booth was equipped with a Samsung Galaxy View 18 inch tablet (Samsung Group, Seoul, South Korea) to render the packaging concept as images (Figure 1) and the biosensory application (University of Melbourne, Melbourne, VIC, Australia), which is able to display the sensory questionnaire and, at the same time, record videos of the participants while observing the stimuli [36]; and an eye-tracking device (GP 3 HD, Gaze point Research Inc., Vancouver, Canada). The eye trackers required that the users sit 30–45 cm from the device [37] for accurate data acquisition. 

The Gazepoint V4.2.0 software (Gaze point Research Inc., Vancouver, Canada) was used to display the packaging samples as joint photographic expert group (.jpeg) images. The TeamViewer v12 software (TeamViewer GmbH, Göppingen, Germany) was used to display the eye-tracking software in the android tablets and allowed panelists to assess the packaging concepts. Instructions were added to the eye-tracking slide show after each packaging sample, to prompt panelists to switch to the biosensory application to answer the sensory questionnaire using a wireless keyboard and mouse. Then, they were asked to return to the TeamViewer app to assess the next packaging concept, and this process was repeated for every sample [38]. Each packaging concept was displayed for a total of 10 seconds, allowing for the assessment of emotional response from video acquisition from panelists [39]. The packaging areas of interest (AOIs) selected were brand name, logo, image, net weight, country of origin (COO) logo, nutritional information, manufacturer’s information, bar code, ingredients, brand name on the back, Fairtrade logo, calorie value, and description and images on the back.

The eye-tracking device was calibrated for every user before presenting them the packaging stimuli using the Gazepoint control. The self-report questionnaire consisted of 15 cm nonstructured continuous scales (Table 1). Perceptual maps were developed using the mean of the responses from panelists to understand where the packaging concepts were positioned based on the valence (not positive/highly positive, x-axis) vs. arousal (calm/excited, y-axis) scores and the reserved (private, self-contained)/unreserved (open/extrovert, y-axis) vs. group (sharing)/solo (consuming alone, x-axis) [40].

### 2.4. Facial Expressions and Eye-Tracking Measurements

Each video was analyzed using FaceReader™. Two different models were used for the facial expression analysis: An East Asian model for the Asian participants and a general model for non-Asians, as recommended by the software manufacturer [30].

Each emotion (Table 2) was averaged and summed for each video from panelists; this value was taken as 100%, then the percentage of each emotion was calculated using Equation (1):

(1) percentage of emotion =(average emotion )/(sum of all emotions)

For the two emotional dimensions and head orientation movements the maximum value was used, whereas, for the facial states, the mean values were obtained due to the nature of the data, which, as previously explained, was transformed to 0 and 1. The eye-tracking data were gathered and processed using the Gazepoint analysis software. The number of fixations is related to the number of times that the gaze goes to the same spot within the AOI and is automatically calculated by the software. Finally, heatmaps were developed to obtain the gaze patterns on each packaging concept qualitatively.

### 2.5. Statistical Analysis

Statistical analyses were performed using Minitab^®^ 18.1 software (Minitab, Inc., State College, Pennsylvania, USA). The self-reported responses, FaceReader™ outputs, and the eye-tracking responses were analyzed for significant differences using one-way analysis of variance (ANOVA, α = 0.05), Tukey simultaneous test to find significant differences, and Pearson correlations (*r*) were also conducted. The eye-tracking results were used to identify the AOI fixated by panelists at a particular time. Then, the facial expressions during this fixation time on each AOI were analyzed using the FaceReader™ results by matching the time of fixation on an AOI with the FaceReader™ results. These measurements were further used for statistical and multivariate analysis. Principal components analysis (PCA) and correlation matrix (CM; *p*-value < 0.05) were performed for all data collected from the self-reported responses, FaceReader™ outputs, along with the total number of fixations using a customized code written in Matlab^®^R2019a (Mathworks Inc., Natick, MA, USA) to assess parameter relationships, data patterns, and significant correlations.

## 3. Results

### 3.1. Perceptual Maps

**Figure 2:**
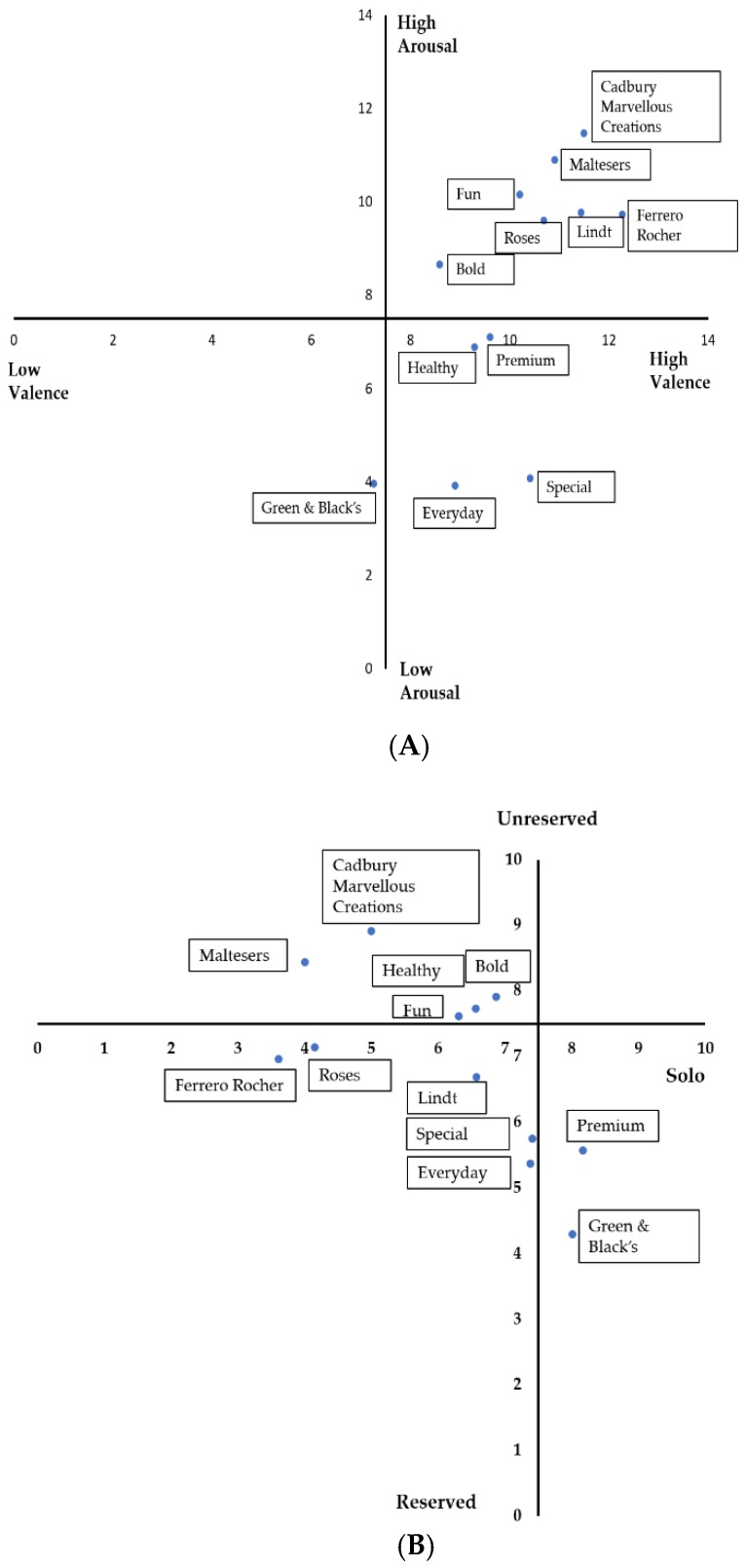
Perceptual map of (**A**) valence vs. arousal (**B**) reserved/unreserved vs. group/solo scores obtained by the packaging concepts showing the positioning of familiar and novel (nonfamiliar) brands.

Consumer scores on valence vs. arousal and reserved/unreserved vs. group/solo scores are shown in the form of perceptual maps (Figure 2A and Figure 2B, respectively). On the basis of the valence-arousal perceptual map, the familiar brands were positioned on the high arousal/high valence quadrant, while the novel (nonfamiliar) brands, except for bold and fun, were positioned in the high valence/low arousal quadrant. From the results shown in Figure 2B, familiar products with multiple pieces (Ferrero Rocher, Roses, and Maltesers) were positioned towards the group axis, while premium concepts (novel: Premium, Special and familiar: Lindt and Green & Black’s) irrespective of familiarity were positioned towards the reserved axis.

### 3.2. Self-Reported Responses for Novel and Familiar Chocolate Packaging

Table 3 shows the mean values and standard deviation (SD) for all self-reported responses measured in novel and familiar packaging concepts. Ferrero Rocher showed the highest value for familiarity (13.7), while all novel concepts showed significantly lower familiarity values as compared with Ferrero Rocher. Green & Black’s (6.03) and Roses (9.19) from the familiar packages also expressed significantly lower familiarity scores. The highest liking score was obtained by Ferrero Rocher (11.9). All novel concepts, Maltesers (9.55) and Green & Black’s (6.39) obtained significantly lower scores for liking.

**Figure 3:**
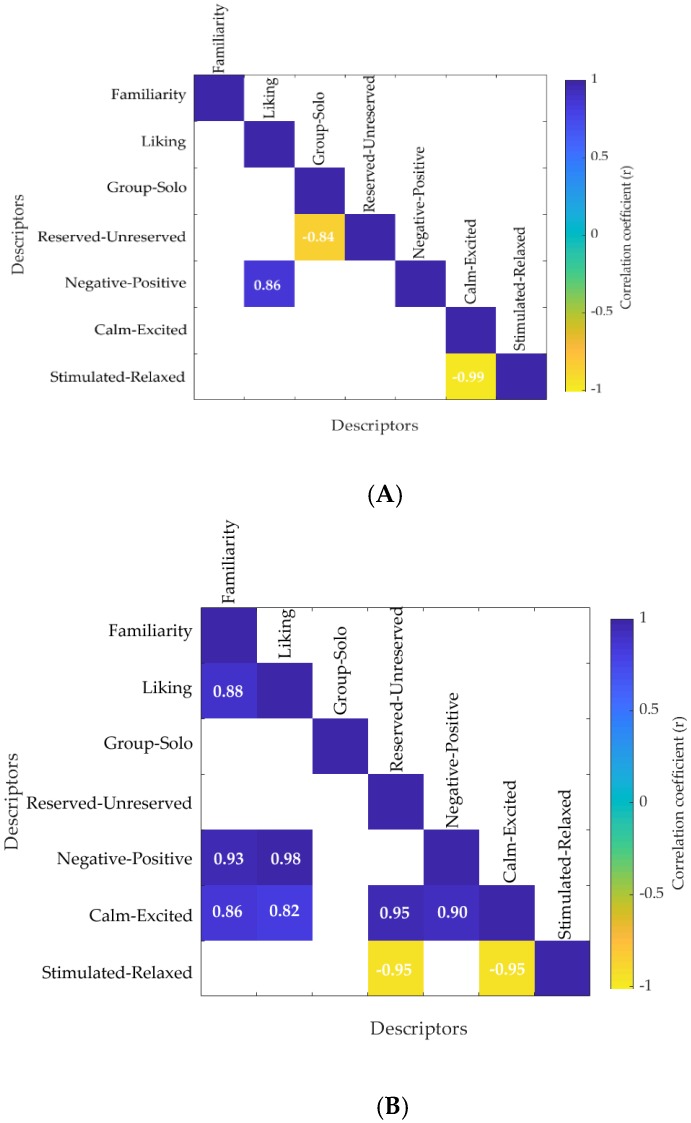
Correlation matrix showing the relationship between self-reported responses in (**A**) novel and (**B**) familiar packaging concepts. Only significant correlations are presented (*p* < 0.05). The color bar represents the correlation coefficients in a scale from –1 to 1, where the blue end denotes the positive correlations and the yellow end represents the negative correlations.

The correlation matrix for the self-reported responses from novel and familiar packaging is shown in Figure 3A and Figure 3B, respectively. Results showed that familiar packaging had a positive correlation between familiarity and liking (*r* = 0.88), while novel packaging did not show any correlation. The familiarity and liking of familiar packaging were also positively correlated with how negative to positive the sample was (*r* = 0.93 and *r* = 0.98) and the level of calmness and excitement of the packaging (*r* = 0.86 and *r* = 0.82). The liking of novel packaging was positively correlated with how negative to positive the sample was (*r* = 0.86). The stimulated/relaxed in familiar packaging was negatively correlated with reserved/unreserved (*r* = −0.95) and calm/excited (*r* = −0.95). A negative correlation was observed in novel packaging between reserved/unreserved with group/solo (*r* = −0.84) and stimulated/relaxed with calm/excited (*r* = −0.99) at *p* = 0.05 level of significance.

### 3.3. Eye-Tracking Measurements

**Figure 4:**
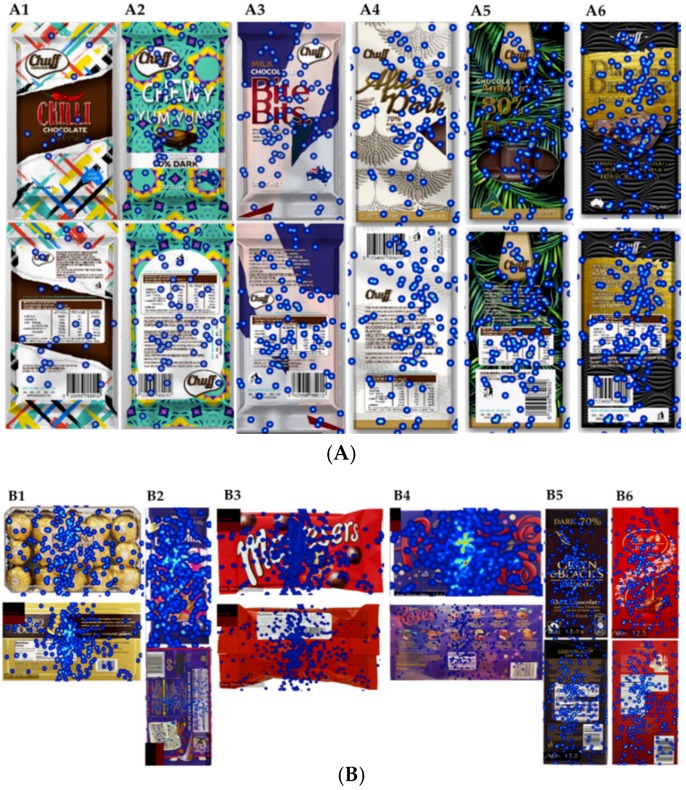
Heatmaps of (**A**) novel and (**B**) familiar packaging concepts for front and back packages: red, the highest number of fixations; orange, second highest number of fixations; yellow, third highest number of fixations; blue, least number of fixations. (**A**) (A1) bold, (A2) fun, (A3) every day, (A4) special, (A5) healthy, and (A6) premium. (**B**) (B1) Ferrero Rocher, (B2) Cadbury Marvellous Creations, (B3) Maltesers, (B4) Roses, (B5) Green & Black’s, and (B6) Lindt.

Heatmaps developed from the average number of fixations of all participants for the novel and familiar packaging concepts are shown in Figure 4A and Figure 4B, respectively. The fixations are more concentrated on the center point in the bold concept (Figure 4A1), whereas, it is more distributed across the packaging in the fun concept (Figure 4A2). Relatively high numbers of fixations were found in the nutritional information (5.3) and the description (5.5) in the very day concept as compared with other AOIs. The texture of the packaging material in the special concept obtained more fixations, whereas, in the healthy concept it was more towards the window of the packaging and the claim ”antioxidants”, and for the premium concept it was on the central image of the rich chocolate with nuts and berries.

Relatively higher numbers of fixations were obtained for the brand name (7.28) and gold color areas of the packaging in the Ferrero Rocher. The center of the packaging presented more fixations in Maltesers (10.7) and Roses (13.4) as compared with the Green & Black’s which was more on the brand name (5.1) and the call out ”organic” (4.35). Fewer fixations were obtained for the Fairtrade logo (0.8). The center (9.65) and the swirly symbol (14.74) in the Lindt sample presented more fixations as compared with the chef on the back of the packaging. However, the fixation points are highly specific to areas and not distributed as zones of focus. As a result, very precise conclusions were not able to be drawn from the heatmap itself. A quantitative analysis of the number of fixations was conducted to obtain more precise results.

The mean values and SD of FaceReader™ outputs (facial expressions) during the fixation for a given AOI in the novel and familiar concepts are shown in Table 4 and Table 5, respectively. There were no significant differences (*p* > 0.05) in the facial expressions during the fixation on all defined AOI when evaluating familiar packaging concepts. However, the emotion “sad” from FaceReader™ resulted in a significantly lower score (0.09) when fixating on the brand name and higher score (0.40) when fixating on the bar code in novel concepts. All other facial expressions obtained nonsignificant differences (*p* > 0.05) during the fixation on other AOIs in the novel packaging concepts.

### 3.4. Multivariate Data Analysis

#### 3.4.1. Eye-Tracking Data

**Figure 5:**
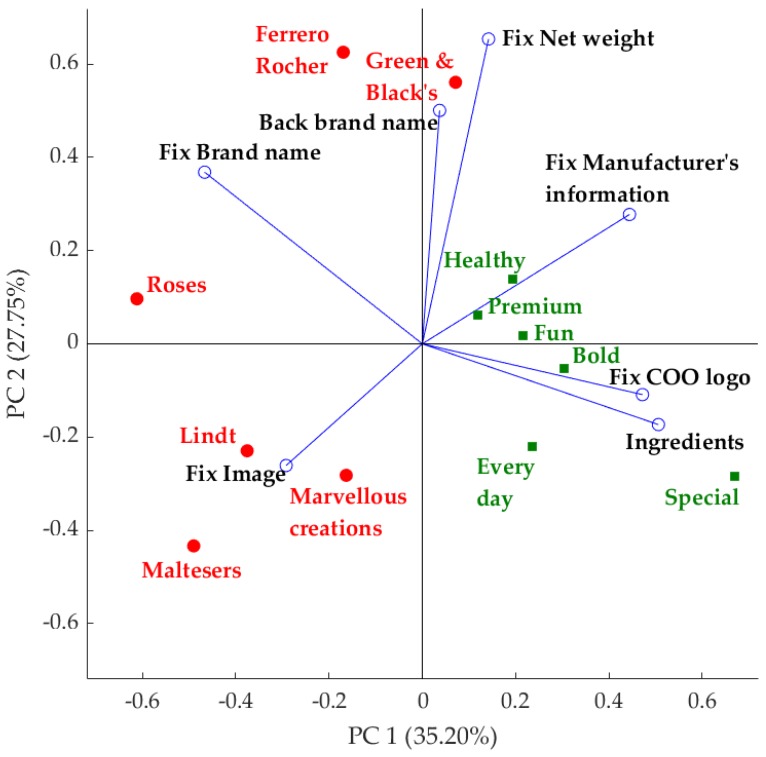
Results from multivariate data analysis: Principal component analysis of the number of eye-tracking fixations (Fix) on AOIs against the packaging concepts. Red/circle = familiar packaging concepts and green/square = novel packaging concepts. The x-axis represents the principal component 1 (PC1) while the y-axis represents the principal component 2 (PC2). Abbreviations: COO, country of origin.

The PCA (Figure 5) obtained for the eye-tracking fixations on AOIs against the packaging concepts explained a total of 62.95% of data variability (PC1 = 35.20% and PC2 = 27.75%). When considering the PCA, a positive relationship was observed between the number of fixations on brand name, image, and back brand name with the familiar packages. On the other hand, consumers tend to fixate more on the nutritional information, manufacturer’s information, ingredients, date of expiry, COO (country of origin) logo and bar code in the novel (nonfamiliar) packaging concepts. The PCA factor loadings (FL, Table S1) show that PC1 was mainly represented by the number of fixations on ingredients (FL = 0.50) and COO logo (FL = 0.47) on the positive side, with special concept being the most representative sample; and brand name (FL = −0.46) and image (FL = −0.29) on the negative side, with Roses being the most representative sample. On the other hand, the PC2 is mainly represented by the net weight (FL = 0.65) and back brand name (FL = 0.50) on the positive side, with Ferrero Rocher being the most representative sample; and image (FL = −0.26) and ingredients (FL = −0.17) on the negative side with Maltesers being the most representative sample. The PC2 separates novel packaging concepts from familiar concepts.

#### 3.4.2. Linking Eye-Tracking Data with FaceReader™ Responses

**Figure 6:**
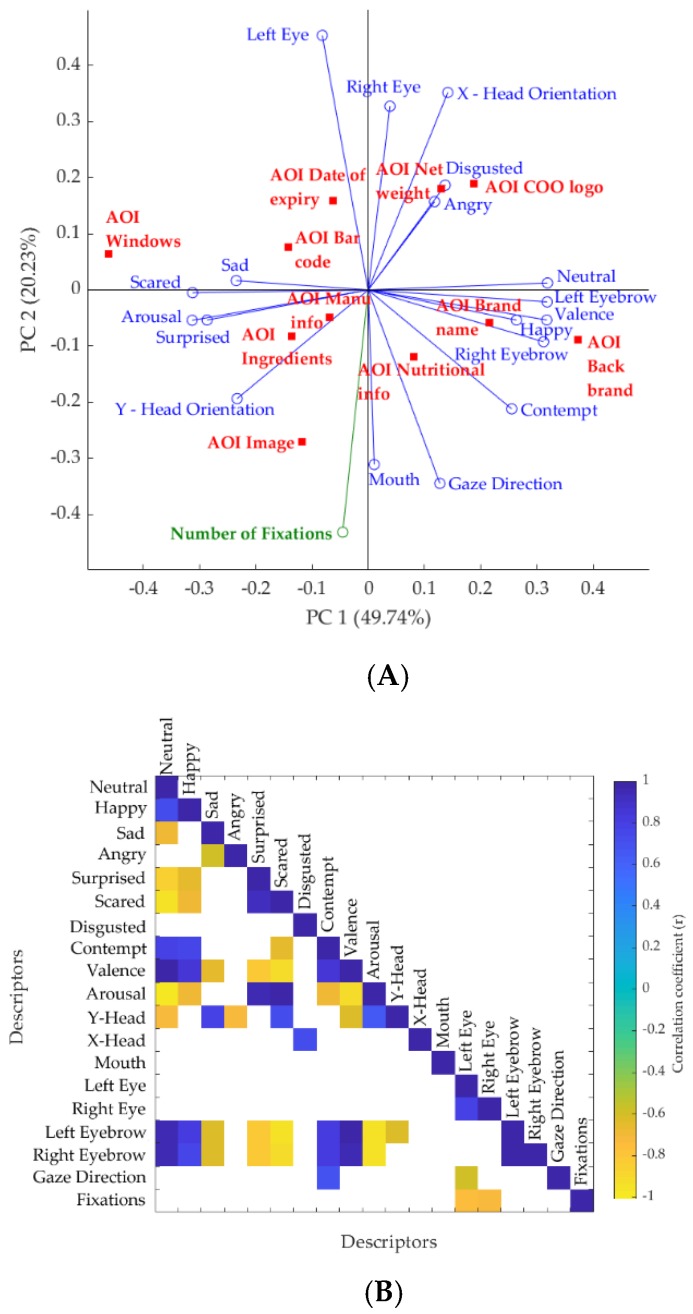
Results of (**A**) principal component analysis (PCA) and (**B**) correlation matrix of the FaceReader™ (FR) outputs and number of fixations from eye tracking against the area of interests (AOIs) of novel packaging concepts. Red/square: AOI, green vector: fixations from eye tracking, blue vector: FR outputs. The x-axis represents the principal component 1 (PC1), and the y-axis represents the principal component 2 (PC2) in the PCA. Abbreviations: AOI, area of interest; COO country of origin; Manu info, manufacturer’s information.

**Figure 7:**
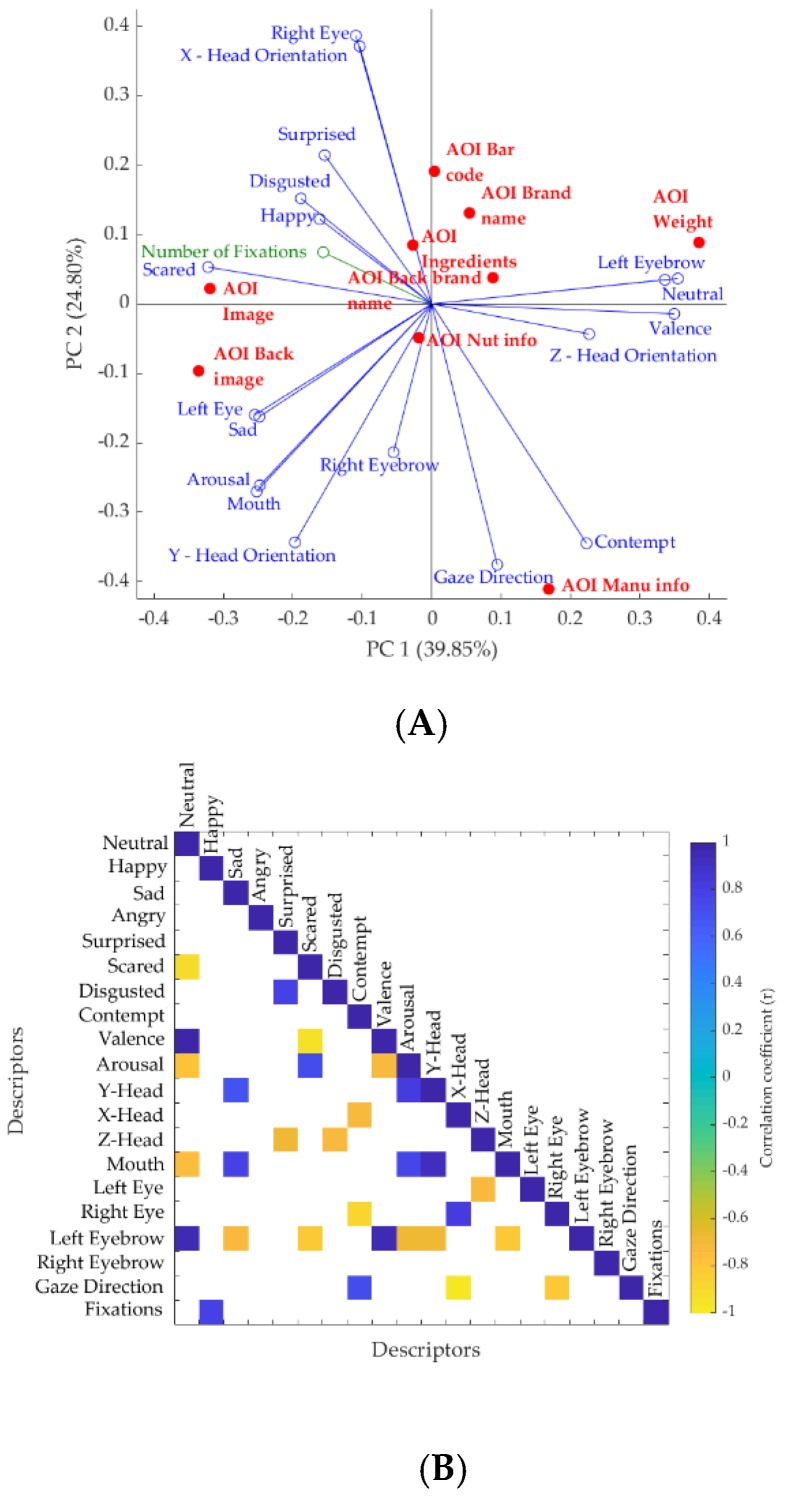
Results of (**A**) principal component analysis (PCA) and (**B**) correlation matrix of the FaceReader™ (FR) outputs and fixations from eye tracking against the area of interests (AOIs) of familiar packaging concepts. Red/square: AOI, green vector: fixations from eye tracking, blue vector: FR outputs. The x-axis represents the principal component 1 (PC1), and the y-axis represents the principal component 2 (PC2) in the PCA. Abbreviations: AOI, area of interest; Manu info, manufacturer’s information; and Nut info, nutritional information.

The PCA and CM obtained for the FaceReader™ (FR) outputs, and total number of fixations (sum of all fixations of all AOIs) from eye tracking against the AOIs of novel and familiar packaging concepts are shown in Figure 6 and Figure 7, respectively. The results showed that the PCA explained a total of 70.0% (PC1 = 49.7% and PC2 = 20.2%) of data variability for novel packaging concepts and 64.7% (PC1 = 39.9% and PC2 = 24.8%) for the familiar packaging samples. On the basis of the PCA, the fixations were associated with the image of the packaging in both novel and familiar concepts. It was observed that “sad” emotion was significantly different for the different AOIs (Table 4).

From the PCA factor loadings for novel chocolate packaging (Table S2) it is shown that PC1 is mainly represented by neutral (FL = 0.31) and valence (FL = 0.31) on the positive side, with back brand name being the most representative AOI; and scared (FL = −0.31) and arousal (FL = −0.31) on the negative side, with the window of the packaging being the most representative AOI. On the other hand, the PC2 is mainly represented by the left eye (FL = 0.45) and X-head orientation (FL = 0.35) on the positive side with net weight being the most representative; and AOI and number of fixations (FL = −0.43) and gaze direction (FL = −0.34) on the negative side, with image as the most representative AOI. It is observed from the PCA factor loadings for familiar chocolate packaging (Table S3) that PC1 is mainly represented by neutral (FL = 0.35) and valence (FL = 0.34) on the positive side, with net weight being the most representative AOI; and scared (FL = −0.32) and arousal (FL = −0.24) on the negative side, with back image being the most representative AOI. On the other hand, the PC2 is mainly represented by the right eye (FL = 0.39) and X-head orientation (FL = 0.37) on the positive side, with bar code being the most representative; and AOI gaze direction (FL = −0.37) and contempt (FL = −0.34) on the negative side, with manufacturer’s information being the most representative AOI.

According to the correlation matrix in novel packaging (Figure 6B), left and right eyebrow from FaceReader™ were positively correlated with valence (*r* = 0.98 and *r* = 0.96), happy (*r* = 0.81 and *r* = 0.77), and neutral (*r* = 0.96 and *r* = 0.95), however, negatively correlated with negative emotions such as sad (*r* = −0.62 and *r* = −0.62), scared (*r* = –0.9 and *r* = −0.93), and surprised (*r* = −0.84 and *r* = −0.83). While the fixations were not correlated with any emotion, they were negatively correlated with the left eye (*r* = −0.75) and right eye (*r* = −0.73) at *p* = 0.05 level of significance.

According to the correlations in familiar packaging (Figure 7B), left eyebrow from participants was positively correlated with neutral (*r* = 0.96) and the dimension valence (*r* = 0.94), however, negatively correlated with negative emotions such as sad (*r* = −0.73) and scared (*r* = −0.83). There was a positive correlation (*r* = 0.78) between fixations and the happy emotion at *p* = 0.05 level of significance.

#### 3.4.3. Integrating Self-Reported and Biometric Responses of Consumers

**Figure 8:**
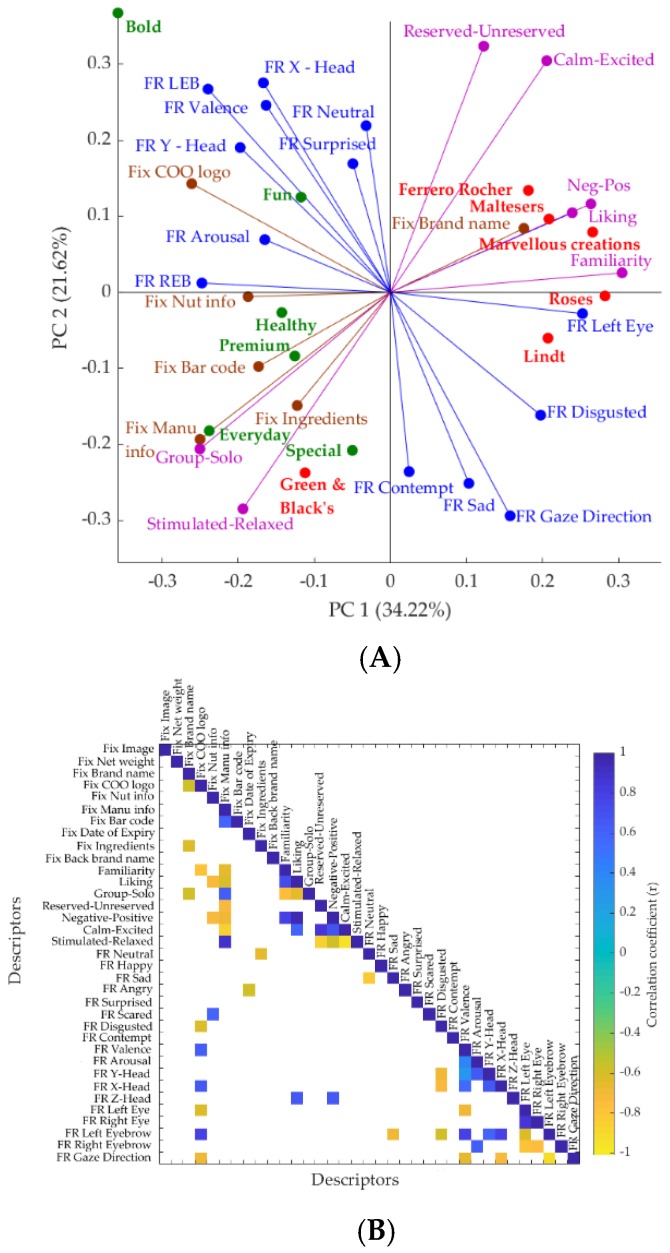
Results of (**A**) principal component analysis (PCA) and (**B**) correlation matrix of all self-reported and biometric responses against the chocolate packaging concepts. Red/circle: familiar packages, green/circle: novel packages, blue vector: fixations from eye tracking, brown vector: FR outputs, purple vector: conscious responses. The x-axis represents the principal component 1 (PC1) while the y-axis represents the principal component 2 (PC2) in the PCA. Abbreviations: AOI, area of interest; FR, FaceReader™; Fix, number of fixations; COO, country of origin; Manu info, manufacturer’s information; Nut info, nutritional information; LEB, left eyebrow; REB, right eyebrow; Neg-Pos, negative to positive.

The PCA and CM obtained for all self-reported and biometric responses are shown in Figure 8. The results from PCA (Figure 8A) showed that the first two principal components (PCs) accounted for a total of 55.84% (PC1 = 34.22% and PC2 = 21.62%) of data variability. On the basis of the PCA, liking, familiarity, and the number of fixations on brand name were correlated with familiar chocolate packaging. The number of fixations on information, ingredients, and barcode, from eye-tracking data, and “surprised” and “neutral” emotions from FaceReader™, were correlated with novel chocolate packaging. It is observed from the PCA factor loadings (Table S4) that PC1 was mainly represented by familiarity (FL = 0.30) and how negative to positive the participants feel about the packaging was (FL = 0.26) on the positive side, and COO logo (FL = −0.26), manufacture’s information (FL = −0.25) and how group−solo the package was (FL = −0.25) on the negative side. On the other hand, the PC2 was mainly represented by how reserved to unreserved the packaging was (FL = 0.32) and how calm to excited the participants were when looking at the packaging sample (FL = 0.30) on the positive side and gaze direction (FL = −0.29), and how stimulated to relaxed the participants were when looking at the packaging sample (FL = −0.28) on the negative side.

According to the correlation matrix (Figure 8B), liking was positively correlated with Z-head orientation (*r* = 0.65), how negative to positive emotions the participants felt when looking at the packaging sample was (r = 0.96) and how calm to excited the participants were when looking at the packaging sample (r = 0.60). The number of fixations on the COO logo was negatively correlated with gaze direction (*r* = −0.69), left eye (*r* = −0.61), and the emotion disgusted (*r* = −0.62) from FaceReader™ outputs and positively correlated with left eyebrow (*r* = 78), X-head (*r* = 0.66) and valence (*r* = 0.63). The number of fixations on the manufacturer’s information was positively correlated with how stimulated to relaxed the participants were when looking at the packaging sample (*r* = 0.87) and how group to solo the participants felt the packaging sample represented (*r* = 0.62) from the conscious responses, and negatively correlated with how calm to excited (*r* = −0.84), how negative to positive emotions (r = −0.68), and how reserved to unreserved the participants were when looking at the packaging sample (*r* = −0.70). Furthermore, familiarity (*r* = −0.66) and liking (*r* = −0.63) were also significant. The number of fixations on nutritional information was positively correlated with “scared” from FaceReader™ (*r* = 0.59) and negatively correlated with how negative to positive emotions the participants felt when looking at the packaging sample (*r* = −0.73) and liking (−0,73). There were negative correlations between the number of fixations on ingredients and “neutral” emotion from FaceReader™ (*r* = −0.64) and between the number of fixations on date of expiry and “angry” emotion from FaceReader™ (*r* = −0.58) at *p* = 0.05 level of significance.

## 4. Discussion

The main finding of this study was that consumer eye fixations, along with emotional responses, could be used in parallel to obtain valuable information to help understand consumer behavior and emotional interpretation of specific AOIs from the packaging. Importantly, the eye fixations on chocolate packages are not always correlated with emotions as they can be affected by their familiarity. The number of fixations on familiar packaging, when considering all AOIs, was positively correlated with the “happy” emotion elicited in people, whereas, the number of fixations on novel packaging was not correlated with any emotions in the participants. However, the “sad” emotion elicited in the participants was significantly higher for fixations on bar code and lower for brand name as compared with other AOIs during the evaluation of novel packaging concepts.

### 4.1. Perceptual Maps

The positioning of familiar chocolate brands in the high valence/high arousal quadrant in the perceptual map (Figure 2A) explains that the panelists were positive and excited about the familiar brands, whereas, they were not likely to be excited about the novel brands. However, bold and fun packaging in the novel concepts have been positioned with the familiar concepts. As explained by Schauss [41], this positioning may be due to the high-wavelength colors of the packaging such as red, orange, and yellow, which are more exciting and arousing than low-wavelength colors. This shows that the bold and fun chocolate packaging concepts were positioned within the opportunity/new gap area to enter into the market when creating new brands/products to engage the consumers emotionally.

The premium packaging from the novel concepts and Green & Black’s from the familiar concepts were positioned in the solo/reserved quadrant (Figure 2B). The black/dark packaging color has been considered as more premium. This is in accordance with the findings from Garber et al. [36] showing that black is related to luxury/premium products while green is related to healthy/organic/ecological products.

### 4.2. Self-Reported Responses

The correlation matrix (Figure 3) and statistical analysis (Table 3) of the self-reported responses showed that the familiar packaging concepts were highly correlated with liking and obtained significantly higher scores for familiarity and liking. These results are in accordance with Birch and Anzman-Frasca [37], who found that familiarity and learning can be used to promote liking. The higher familiarity scores obtained for familiar brands are in accordance also with Dahlén et al. [42] who found that familiar brands have better recall when compared to novel brands. This was also shown in the PCA (Figure 8A) and correlation matrix (Figure 8B) constructed using all conscious and unconscious responses, where the “happy” emotion, liking and familiarity, and fixations on brand name and image were all correlated with familiar chocolate packaging. The SD values of the self-reported responses were high, which is in accordance with another study conducted using visual stimuli with high SD scores for self-reported responses [43]. The high variability found in the responses might be due to cultural differences. Hence, further studies with a higher number of participants and from different cultures (Asian and non-Asian) would aid in the reduction of the dispersion of the data and a better understanding of how different consumers respond to packaging concepts. The CM showed that the liking score of self-reported responses was positively correlated with Z-head orientation, which explains that the consumers had approached the screen when they liked the packaging. This is in accordance of the findings of Seibt et al. [44] who stated that positive stimuli facilitate behavior for either approaching the stimulus (object as reference point) or for bringing the stimulus closer (self as reference point) and negative stimuli facilitate behavior for withdrawing from the stimulus or for pushing the stimulus away.

### 4.3. Eye-Tracking Measurements

Heatmaps were a very effective way to represent eye-tracking data since they provide an overall view of the gaze activity of the respondents evaluating the packaging. High fixations on the texture of the packaging material in the special concept explain the value of texture of chocolate packaging. This has been further confirmed by Yu [45], who found that the use of shading and texture can add details to an image and provide overall surface quality.

The AOIs separation provided an opportunity to obtain quantitative measurements [21], which aided in the study of the emotional responses of consumers while fixating on AOIs in novel and familiar packaging concepts. While there were no significant differences in emotional responses among the AOIs in familiar concepts, there were significant variations in the emotion “sad” from FaceReader™ while fixating the AOIs in novel concepts. Further research in this area may be conducted by comparing these physiological with self-reported responses on emotion levels obtained consciously.

### 4.4. Multivariate Data Analysis

The higher number of fixations on brand name for familiar brands found in this study (Figure 5) supports the explanation of Deliza and MacFie [46] that the brand name is regarded as a very useful element for a product to be selected by consumers among other competing brands. On the other hand, panelists fixated less on the brand name of novel products.

A higher number of fixations on information (nutritional information, COO logo, bar code, DOE, manufacturer’s information, and ingredients) was observed in novel chocolate packaging (Figure 5). Assumptions are made on the foundation that longer eye fixations are associated with higher levels of attention and interest, and greater interest equals positive reaction, liking, and assimilation. However, eye tracking alone cannot provide accurate information in terms of emotional responses from consumers [18]. It has been shown that 7% of messages to other people are conveyed by spoken words, 38% by voice intonation, and 55% by facial expressions [47]. This shows the importance of understanding the reactions of consumers through facial expressions. There have been studies conducted using FR to obtain consumer emotional responses while evaluating food packaging [48], food products [49], problem-solving [50], and evaluating the texture of images [51]. However, there is a lack of studies based on the emotional responses obtained by FR with other responses, such as eye tracking to better understand the visual attention of consumers. Therefore, the main contribution of this study was to establish the link between the visual focus using eye tracking (number of eye fixations) and the emotional responses (facial expressions) of novel and familiar chocolate packaging designs. Specifically, the correlation matrix showed that the fixations on familiar chocolate packages were highly positively correlated with happiness, whereas, the fixations on the novel chocolate packages were not correlated with emotions. Furthermore, it was clear from the results of this study that mere fixations on packaging do not mean that consumers pay attention or are emotionally engaged with the packaging. There could be additional attributes which are important in emotional engagement with products, for example, the sensory characteristics/taste of the product. It is important to conduct further research to understand the emotional engagement of consumers towards food products based on food packaging together with sensory characteristics.

A technical limitation of this study was that, although the eye trackers are low cost, each device requires the use of a computer to run the data acquisition and analysis. This can be solved by integrating the eye tracking to the integrated camera system and the biosensory app using the available software developer kit (SDK) which will be incorporated for future studies. Furthermore, more robust results might be obtained by increasing the sample size (as per the sensory guidelines from the Society of Sensory Professionals). This study did not consider gender effect. However, it will be important in future studies to consider these effects since females are believed to be more expressive and involved in more nonverbal behaviors than men [52].

## 5. Conclusions

Fixations on a specific AOI vary in familiar and novel chocolate packaging. Fixations are not necessarily associated with emotions, especially in novel concepts. However, fixations on familiar chocolate packaging were correlated with the happy emotion. This approach demonstrates the value of integrating eye-tracking data with physiological responses and also how these combined methodologies can contribute to a better understanding of how packaging is evaluated by consumers based on emotional responses. This study would be of interest for the industry as there is no need to use the printed packaging designs, which facilitates the evaluation and redesign of labels and, at the same time, makes it more cost-effective and less time-consuming. These results provide guidelines for chocolate package designers on how they can develop packages to engage consumers with packaging emotionally. It shows that it is important to move a step beyond self-reported responses by incorporating more advanced techniques like biometrics. This approach is relatively new. However, it would allow evaluation of the intrinsic and extrinsic attributes of products which would help to better understand consumer perceptions.

## Figures and Tables

**Table 1:** Questions for self-reported responses and the answer options provided to panelists. The categories were equally spaced in the answer options, and the panelists could mark their response anywhere within the line.

Question	Anchors in Continuous Line Scale
How familiar is the package?	Not at all familiar–somewhat unfamiliar–neither unfamiliar nor familiar–somewhat familiar–extremely familiar
Rate how you like the sample?	Dislike extremely–somewhat dislike–neither like nor dislike–somewhat like–like extremely
How group to solo is the package?	Group–less group–moderate–less solo–solo
How reserved to unreserved is the package?	Reserved–less reserved–moderate–less unreserved–unreserved
How negative to positive is the package?	Negative–less negative–moderate–less positive–positive
How calm to excited is the package?	Calm–less calm–moderate–less excited
How stimulated to relaxed is the package?	Stimulated–less stimulated–moderate–less relaxed–relaxed

**Table 2:** Category, outputs, and scale of FaceReader™ responses.

Category	Output	Scale
Emotions	Neutral	0–1
	Happy	0–1
	Sad	0–1
	Angry	0–1
	Surprised	0–1
	Scared	0–1
	Disgusted	0–1
	Contempt	0–1
Dimensions	Valence	−1–1
	Arousal	0–1
Head orientation	X	Degrees
	Y	Degrees
	Z	Degrees
Gaze direction	Left	−1
	Forward	0
	Right	1
Facial states	Mouth	0 (closed), 1 (opened)
	Left eye	0 (closed), 1 (opened)
	Right eye	0 (closed), 1 (opened)
	Left eyebrow	−1 (lowered), 0 (neutral), 1 (raised)
	Right eyebrow	−1 (lowered), 0 (neutral), 1 (raised)

**Table 3:** Mean values (top) and standard deviation (bottom) of self-reported responses for novel and familiar packaging concepts.

Familiarity	Package Concept	Familiarity	Liking	Group-Solo	Reserved-Unreserved	Negative-Positive	Calm-Excited	Stimulated-Relaxed
Novel	Bold	3.14 ^e^ ± 3.43	8.12 ^c,d,e^ ± 3.01	6.75 ^a,b,c^ ± 3.95	7.78 ^a,b^ ± 3.19	8.69 ^c,d^ ± 3.96	8.75 ^b,c,d^ ± 4.73	6.43 ^c,d^ ± 4.07
	Fun	4.27 ^d,e^ ± 4.03	9.02 ^b,c,d^ ± 3.40	6.41 ^a,b,c,d^ ± 3.75	7.73 ^a,b^ ± 3.55	10.1 ^a,b,c^ ± 3.78	10.1 ^a,b^ ± 3.91	6.16 ^c,d^ ± 4.45
	Every day	5.71 ^c,d^ ± 4.25	7.92 ^d,e^ ± 3.70	7.38 ^a,b^ ± 4.23	5.37 ^b,c^ ± 4.18	8.89 ^c,d^ ± 4.25	3.91 ^e^ ± 4.11	9.55 ^a,b^ ± 3.60
	Special	6.20 ^c,d^ ± 4.37	9.98 ^a,b,c,d^ ± 3.45	7.41 ^a,b^ ± 4.17	5.74 ^b,c^ ± 3.84	10.4 ^a,b,c^ ± 3.52	4.07 ^e^ ± 3.43	9.74 ^a^ ± 3.94
	Healthy	5.59 ^c,d,e^ ± 4.46	9.12 ^b,c,d^ ± 3.58	6.56 ^a,b,c,d^ ± 4.19	7.73 ^a,b^ ± 3.82	9.29 ^b,c,d^ ± 4.07	6.88 ^d^ ± 4.82	7.99 ^a,b,c^ ± 4.03
	Premium	6.95 ^c^ ± 4.57	9.37 ^b,c,d^ ± 3.93	8.16 ^a^ ± 4.76	5.56 ^b,c^ ± 4.56	9.60 ^b,c^ ± 4.18	7.10 ^c,d^ ± 4.89	7.54 ^a,b,c^ ± 4.27
Familiar	Ferrero Rocher	13.7 ^a^ ± 2.33	11.9 ^a^ ± 2.73	3.60 ^e^ ± 4.26	6.95 ^a,b^ ± 4.61	12.3 ^a^ ± 2.58	9.72 ^a,b^ ± 4.48	7.13 ^b,c^ ± 4.92
	Marvellous Creations	11.6 ^a,b^ ± 4.35	10.5 ^a,b^ ± 3.24	5.00 ^b,c,d,e^ ± 4.83	8.90 ^a^ ± 4.24	11.5 ^a,b^ ± 3.43	11.5 ^a^ ± 3.41	4.39 ^d^ ± 4.04
	Maltesers	13.1 ^a^ ± 2.67	9.55 ^b,c,d^ ± 3.84	4.00 ^d,e^ ± 4.56	8.43 ^a^ ± 4.22	10.9 ^a,b,c^ ± 3.41	10. 9 ^a,b^ ± 3.88	4.57 ^d^ ± 3.82
	Roses	9.19 ^b^ ± 4.54	10.1 ^a,b,c^ ± 2.94	4.14 ^c,d,e^ ± 3.99	7.13 ^a,b^ ± 4.23	10.7 ^a,b,c^ ± 3.47	9.59 ^a,b,c^ ± 4.41	6.41 ^c,d^ ± 4.27
	Green & Black’s	6.03 ^c,d^ ± 4.81	6.39 ^e^ ± 3.16	8.01 ^a^ ± 4.66	4.28 ^c^ ± 4.18	7.25 ^d^ ± 3.18	3.95 ^e^ ± 3.53	9.73 ^a^ ± 3.57
	Lindt	12.00 ^a,b^ ± 3.99	10.8 ^a,b^ ± 3.24	6.57 ^a,b,c,d^ ± 4.53	6.69 ^a,b,c^ ± 4.36	11.4 ^a,^ ± 3.10	9.77 ^a,b^ ± 4.62	5.79 ^c,d^ ± 4.29

^a,b,c,d,e^ Values that do not share a letter are significantly different (*p* < 0.05) within columns.

**Table 4:** Mean values (top) and standard deviation (bottom) of FaceReader™ outputs during the fixation on a defined AOI in novel packaging concepts.

Familiarity	AOI	Neutral ^NS^	Happy ^NS^	Sad	Angry ^NS^	Surprised ^NS^	Scared ^NS^	Disgusted ^NS^	Contempt ^NS^	Valence ^NS^	Arousal ^NS^
Novel	Brand name	0.80 ± 0.12	0.08 ± 0.19	0.09 ^a^ ± 0.08	0.15 ± 0.20	0.09 ± 0.12	0.01 ± 0.01	0.005 ± 0.004	0.12 ± 0.14	−0.10 ± 0.07	0.33 ± 0.10
	Image	0.42 ± 0.29	0.01 ± 0.01	0.24 ^a,b^ ± 0.12	0.08 ± 0.07	0.23 ± 0.17	0.38 ± 0.39	0.007 ± 0.005	0.03 ± 0.05	−0.47 ± 0.31	0.59 ± 0.34
	Ingredients	0.44 ± 0.32	0.009 ± 0.01	0.24 ^a,b^ ± 0.09	0.10 ± 0.10	0.22 ± 0.19	0.42 ± 0.39	0.01 ± 0.01	0.03 ± 0.05	−0.50 ± 0.31	0.57 ± 0.36
	Manufacturers information	0.43 ± 0.24	0.02 ± 0.06	0.23 ^a,b^ ± 0.17	0.20 ± 0.25	0.23 ± 0.17	0.34 ± 0.36	0.006 ± 0.005	0.01 ± 0.02	−0.51 ± 0.23	0.56 ± 0.30
	Nutritional information	0.50 ± 0.26	0.06 ± 0.12	0.17 ^a,b^ ± 0.11	0.11 ± 0.11	0.16 ± 0.19	0.26 ± 0.35	0.03 ± 0.05	0.07 ± 0.11	−0.33 ± 0.30	0.54 ± 0.33
	Net weight	0.62 ± 0.22	0.03 ± 0.04	0.18 ^a,b^ ± 0.18	0.14 ± 0.25	0.14 ± 0.26	0.14 ± 0.21	0.02 ± 0.03	0.05 ± 0.31	−0.25 ± 0.23	0.45 ± 0.22
	Bar code	0.42 ± 0.19	0.02 ± 0.04	0.40 ^b^ ± 0.20	0.10 ± 0.08	0.14 ± 0.26	0.30 ± 0.33	0.01 ± 0.009	0.01 ± 0.02	−0.50 ± 0.18	0.54 ± 0.29

a,b values that do not share a letter are significantly different (*p* < 0.05) within column; NS: nonsignificance between the AOIs for a given emotion/ dimension at α = 0.05.

**Table 5:** Mean values (top) and standard deviation (bottom) of FaceReader™ outputs during the fixation on a defined AOI in familiar packaging concepts.

Familiarity	AOI	Neutral ^NS^	Happy ^NS^	Sad ^NS^	Angry ^NS^	Surprised ^NS^	Scared ^NS^	Disgusted ^NS^	Contempt ^NS^	Valence ^NS^	Arousal ^NS^
Familiar	Brand name	0.57 ± 0.31	0.008 ± 0.005	0.19 ± 0.13	0.05 ± 0.04	0.22 ± 0.10	0.28 ± 0.36	0.01 ± 0.01	0.02 ± 0.02	−0.340.31	0.53 ± 0.35
	Image	0.24 ± 0.06	0.01 ± 0.01	0.44 ± 0.30	0.08 ± 0.05	0.17 ± 0.13	0.46 ± 0.33	0.009 ± 0.006	0.001 ± 0.002	−0.64 ± 0.14	0.67 ± 0.45
	Ingredients	0.43 ± 0.34	0.007 ± 0.009	0.21 ± 0.25	0.08 ± 0.04	0.16 ± 0.18	0.39 ± 0.34	0.01 ± 0.007	0.02 ± 0.03	−0.45 ± 0.35	0.65 ± 0.26
	Manufacturers information	0.58 ± 0.38	0.005 ± 0.004	0.27 ± 0.30	0.04 ± 0.02	0.14 ± 0.17	0.15 ± 0.18	0.008 ± 0.007	0.16 ± 0.28	−0.28 ± 0.29	0.75 ± 0.12
	Nutritional Information	0.45 ± 0.27	0.007 ± 0.007	0.17 ± 0.09	0.09 ± 0.05	0.21 ± 0.14	0.38 ± 0.34	0.008 ± 0.005	0.02 ± 0.05	−0.41 ± 0.31	0.67 ± 0.25
	Net weight	0.71 ± 0.30	0.004 ± 0.002	0.11 ± 0.03	0.09 ± 0.08	0.13 ± 0.08	0.12 ± 0.21	0.004 ± 0.004	0.07 ± 0.10	−0.21 ± 0.13	0.48 ± 0.37
	Bar code	0.50 ± 0.37	0.007 ± 0.005	0.16 ± 0.13	0.05 ± 0.03	0.22 ± 0.09	0.44 ± 0.47	0.008 ± 0.006	0.008 ± 0.01	−0.46 ± 0.44	0.61 ± 0.32

## Supplementary Materials

The following are available online at https://www.mdpi.com/2304-8158/8/7/253/s1, Table S1: Factor loadings from the principal components analysis for the descriptors used in the analysis of Figure 5 and for the first two principal components (PC1 and PC2), Table S2: Factor loadings from the principal components analysis for the descriptors used in the analysis of Figure 6 and for the first two principal components (PC1 and PC2), Table S3: Factor loadings from the principal components analysis for the descriptors used in the analysis of Figure 7 and for the first two principal components (PC1 and PC2), Table S4: Factor loadings from the principal components analysis for the descriptors used in the analysis of Figure 8 and for the first two principal components (PC1 and PC2).
