# Supplementary material for: Consumer Acceptability, Eye Fixation, and Physiological Responses: A Study of Novel and Familiar Chocolate Packaging Designs Using Eye-Tracking Devices
Source: Foods. 2019 Jul 12;8(7):253. doi: 10.3390/foods8070253 (PMC6679145; doi:10.3390/foods8070253)
Supplement: Supplementary file 1 [file foods-08-00253-s001.pdf]

Supplementary Materials: The following are available online at www.mdpi.com/xxx/s1,

Table S1: Factor loadings from the principal components analysis for the descriptors used in the analysis of Figure 5 and for the first two principal components (PC1 and PC2).

Descriptor	PC 1	PC 2
Image	-0.29	-0.26
Net weight	0.14	0.65
Brand name	-0.46	0.36
COO logo	0.47	-0.10
Manufacturer's information	0.44	0.27
Ingredients	0.50	-0.17
Back brand name	-0.03	0.50

Table S2: Factor loadings from the principal components analysis for the descriptors used in the analysis of Figure 6 and for the first two principal components (PC1 and PC2)

Descriptor	PC 1	PC 2
Neutral	0.31	0.01
Happy	0.26	-0.05
Sad	-0.23	0.01
Angry	0.11	0.15
Surprised	-0.28	-0.05
Scared	-0.31	-0.05
Disgusted	0.13	0.18
Contempt	0.25	-0.21
Valence	0.31	-0.02
Arousal	-0.31	-0.01
Y-Head orientation	-0.23	-0.19
X-Head orientation	0.14	0.35
Mouth	0.01	-0.31
Left eye	-0.08	0.45
Right eye	0.03	0.32
Left eyebrow	0.31	-0.05
Right eyebrow	0.31	-0.09
Gaze direction	0.12	-0.34

Number of fixations	-0.04	-0.43

Table S3: Factor loadings from the principal components analysis for the descriptors used in the analysis of Figure 7 and for the first two principal components (PC1 and PC2)

Descriptor	PC 1	PC 2
Neutral	0.35	0.04
Happy	-0.15	0.11
Sad	-0.23	-0.17
Angry	-0.13	0.06
Surprised	-0.14	0.20
Scared	-0.32	0.04
Disgusted	-0.17	0.13
Contempt	0.22	-0.34
Valence	0.34	-0.01
Arousal	-0.24	-0.27
Y-Head orientation	-0.19	-0.34
X-Head orientation	-0.11	0.37
Z-Head orientation	0.21	-0.02
Mouth	-0.24	-0.26
Left eye	-0.24	-0.17
Right eye	-0.12	0.39
Left eyebrow	0.33	0.03
Right eyebrow	-0.05	-0.21
Gaze direction	0.10	-0.37
Number of fixations	-0.14	0.06

Table S4: Factor loadings from the principal components analysis for the descriptors used in the analysis of Figure 8 and for the first two principal components (PC1 and PC2)

Descriptor	PC 1	PC 2
Fix Brand name	0.17	0.08
Fix COO logo	-0.26	0.14
Fix Nutritional information	-0.18	-0.01
Fix Manufacture's information	-0.25	-0.19
Fix Bar code	-0.17	-0.09

Fix Ingredients	-0.12	-0.14
Familiarity	0.30	-0.02
Liking	0.23	0.10
Group-Solo	-0.25	-0.20
Reserved-Unreserved	0.12	0.32
Negative-Positive	0.26	0.11
Calm-Excited	0.20	0.30
Stimulated-Relaxed	-0.19	-0.28
FR Neutral	-0.03	0.21
FR Sad	0.10	-0.25
FR Surprised	-0.04	0.16
FR Disgusted	0.19	-0.16
FR Contempt	-0.02	-0.23
FR Valence	-0.16	0.24
FR Arousal	-0.16	0.06
FR Y - Head	-0.19	0.19
FR X - Head	-0.16	0.27
FR Left Eye	0.25	-0.02
FR Left Eyebrow	-0.23	0.26
FR Right Eyebrow	-0.24	0.01
FR Gaze Direction	0.15	-0.29
